# *In vitro* biofilm formation of *Gardnerella vaginalis* and *Escherichia coli* associated with bacterial vaginosis and aerobic vaginitis

**DOI:** 10.3389/fcimb.2024.1387414

**Published:** 2024-05-01

**Authors:** Xiang Shang, Huihui Bai, Linyuan Fan, Xin Zhang, Xiaowen Zhao, Zhaohui Liu

**Affiliations:** ^1^ Department of Gynecology, Beijing Obstetrics and Gynecology Hospital, Capital Medical University, Beijing Maternal and Child Health Care Hospital, Beijing, China; ^2^ Department of Clinical Laboratory, Beijing Obstetrics and Gynecology Hospital, Capital Medical University, Beijing Maternal and Child Health Care Hospital, Beijing, China

**Keywords:** *Gardnerella vaginalis*, *Escherichia coli*, biofilm, mixed vaginitis, bacterial vaginosis, aerobic vaginitis

## Abstract

**Objective:**

To determine the optimum biofilm formation ratio of *Gardnerella vaginalis* (*G. vaginalis*) in a mixed culture with *Escherichia coli* (*E. coli*).

**Methods:**

*G. vaginalis* ATCC14018, *E. coli* ATCC25922, as well as five strains of *G. vaginalis* were selected from the vaginal sources of patients whose biofilm forming capacity was determined by the Crystal Violet method. The biofilm forming capacity of *E. coli* in anaerobic and non-anaerobic environments were compared using the identical assay. The Crystal Violet method was also used to determine the biofilm forming capacity of a co-culture of *G. vaginalis* and *E. coli* in different ratios. After Live/Dead staining, biofilm thickness was measured using confocal laser scanning microscopy, and biofilm morphology was observed by scanning electron microscopy.

**Results:**

The biofilm forming capacity of *E. coli* under anaerobic environment was similar to that in a 5% CO_2_ environment. The biofilm forming capacity of *G. vaginalis* and *E. coli* was stronger at 10^6^:10^5^ CFU/mL than at other ratios (*P*<0.05). Their thicknesses were greater at 10^6^:10^5^ CFU/mL than at the other ratios, with the exception of 10^6^:10^2^ CFU/mL (*P*<0.05), under laser scanning microscopy. Scanning electron microscopy revealed increased biofilm formation at 10^6^:10^5^ CFU/mL and 10^6^:10^2^ CFU/mL, but no discernible *E. coli* was observed at 10^6^:10^2^ CFU/mL.

**Conclusion:**

*G. vaginalis* and *E. coli* showed the greatest biofilm forming capacity at a concentration of 10^6^:10^5^ CFU/mL at 48 hours and could be used to simulate a mixed infection of bacterial vaginosis and aerobic vaginitis *in vitro*.

## 1 Introduction

Mixed vaginitis is a group of vaginal infections caused by the coexistence of two or more vaginal pathogens, leading to abnormalities in the vaginal microenvironment and causing different signs and symptoms (Paavonen and Brunham, 2018; Xiao et al., 2022). Depending on the vaginal pathogen, they can be further categorized into more than 10 types. The most common of which include bacterial vaginosis (BV), trichomoniasis vaginalis (TV), vulvovaginal candidiasis (VVC), and aerobic vaginitis (AV).

Mixed vaginitis is becoming more common (Maladkar, 2015). The literature reports varying incidence rates across different regions. This variation may be attributed to various factors such as varying levels of pathogen exposure among different races and regions, atypical clinical symptoms, significant cognitive differences, and variations in detection methods and levels (Nyirjesy et al., 2020). High-quality epidemiological data is not yet available, and the incidence varies considerably domestically and internationally, ranging from 4.44% to 56.80% (Rivers et al., 2011; Wang et al., 2016). Depending on the vaginal pathogens, they can be divided into various types, such as BV + AV, BV + VVC, BV + TV, and VVC+AV. According to earlier reports, the most common kind of mixed vaginitis is BV + VVC. Conversely, since the introduction of the concept of AV by Donders et al. in 2002 (Donders et al., 2002) and the increased awareness of AV among clinicians in recent years, the prevalence of BV + AV is currently considered to be the highest among the types of mixed vaginitis (Salinas et al., 2020; Pacha-Herrera et al., 2022), ranging from 37.14% to 55.34% (Fan et al., 2013; Cooperative Group of Infectious Disease, Chinese Society of Obstetrics and Gynecology, Chinese Medical Association, 2021). Both BV and AV can result in serious health complications for women. In addition to the common symptoms of vaginitis, they can also lead to a higher likelihood of pelvic inflammatory disease and HPV infection. When present during pregnancy, the risks of miscarriage, preterm labor, chorioamnionitis, neonatal infections, and other adverse outcomes increase significantly (Feng et al., 2023; Kenfack-Zanguim et al., 2023; Qulu et al., 2023).

The mechanism of mixed vaginitis is unclear, but mixed infections with multiple pathogens often result in the formation of a mixed biofilm that can interact in a variety of ways, either synergistic or antagonistic interactions. This increases the pathogens’ resistance and capacity to elude the host’s immune response (Allison et al., 2016; Castro et al., 2016; Koo et al., 2018; Lohse et al., 2018). Machado et al (MaChado and Cerca, 2015; Salinas et al., 2018) pointed out that the biofilm formation by *Gardnerella vaginalis* (*G. vaginalis*) and other anaerobic bacterial plays a key role in the pathogenesis of BV. Therefore, the interaction and influence between multiple pathogens in mixed vaginitis should be clarified during treatment to further adjust the usage and dosage of applicable medications to improve the curative effect. However, studies on mixed infections, especially those involving interspecies interactions between multiple pathogens and the impact of biofilms on diseases, are still at early stages (Schlecht et al., 2015). Therefore, there is an urgent need to study the biofilm state and the interaction between different pathogens in mixed infections and to construct an infection model of mixed vaginitis. This will provide ideas for improving the efficacy of the treatment of mixed vaginitis and reducing the risk of recurrence.

While a number of studies have examined the relationship between *G. vaginalis* and *Candida* in BV + VVC in mixed vaginitis (Fox et al., 2014; Allison et al., 2016); relatively few have examined BV + AV (Janulaitiene et al., 2017; Morrill et al., 2020). *G. vaginalis* is predominant in BV (Salinas et al., 2018), and *Escherichia coli* (*E. coli*) is also predominant in patients with AV (Wang et al., 2020; Prasad et al., 2021). Therefore, this study aimed to establish a stable *in vitro* situation that is most suitable for the growth of *G. vaginalis* and *E. coli*, to simulate the mixed infection status of BV and AV. Additionally, it provided a basic *in vitro* model for the subsequent study of the related mechanism and treatment of BV + AV mixed infection.

## 2 Materials and methods

### 2.1 Collection of specimens

The strains of *Gardnerella* and *E. coli* in our study included *G. vaginalis* ATCC14018 (Vaneechoutte et al., 2019), *E. coli* ATCC25922, and other *Gardnerella* species isolated from patients with BV. Clinical symptoms and the Nugent score were used to diagnose BV in patients who did not have any other urogenital infections. Antibiotics had not been given to the patients during the preceding week. This study was approved by the Ethics Committee of Beijing Obstetrics and Gynecology Hospital, Capital Medical University (2022-KY-064-01).

### 2.2 Biofilm formation by the crystal violet method

*G. vaginalis* and *E. coli* were cultured using supplementing with brain-heart infusion (sBHI), which comprised brain-heart infusion broth (AOBOX, Beijing, China), 0.3% starch, and 0.3% glucose (He et al., 2021). The overnight growth of *G. vaginalis* and *E. coli* were adjusted to 0.5 McFarland (1.5 × 10^8^ CFU/mL), and diluted to 1 ×10^6^ CFU/mL, and then added to a 96-well plate (Falcon, Corning Inc., NY, USA). Then it was incubated at 37°C in an anaerobic environment (AnaeroPouch-Anaero, C-1, Mitsubishi Gas Chemical CO., INC., Tokyo, Japan). After incubation, the bacterial solution was discarded and the wells washed by 1× phosphate buffer saline (PBS) (P1020, Solarbio, Beijing, China). After staining the samples with 0.2 percent crystal violet (C8470, Solarbio, Beijing, China), they were rinsed with 1× PBS and subsequently decolored with 95% alcohol. The OD value of the eluate was detected using an enzyme marker at 580 nm. The OD cutoff value (ODc) was determined by taking the mean value of the negative controls that contained only sBHI plus three times the standard deviation. The biofilm forming capacity was then calculated by dividing the microtiter wells’ OD value by ODc (Shiroda and Manning, 2020; Dantas-Medeiros et al., 2021). All analyses were repeated three times on different dates.

#### 2.2.1 Comparison of the biofilm forming capacity of *E. coli* in anaerobic and non-anaerobic environments

*G. vaginalis* is an anaerobic bacterium that requires stringent anaerobic environments. On the other hand, *E. coli* is a facultative anaerobic bacterium that can be cultured under favorable conditions. It is necessary to conduct preliminary experiments to confirm whether the biofilm forming capacity of *E. coli* differs in anaerobic and non-anaerobic environments. The biofilm forming capacity of *E. coli* was determined by the Crystal Violet (CV) method after incubation at 37°C, 5% CO_2_ and anaerobic environment for 48 hours.

#### 2.2.2 Comparison of the biofilm forming capacity of *G. vaginalis* alone, *E. coli* alone, and both when co-cultured for 48 hours.

*G. vaginalis* ATCC14018 and *E. coli* ATCC25922 were mixed and cultured in sBHI to formulate bacterial volume ratios of 10^0^:10^6^, 10^1^:10^6^, 10^2^:10^6^, 10^3^:10^6^, 10^4^:10^6^, 10^5^:10^6^, 10^6^:10^6^, 10^6^:10^5^, 10^6^:10^4^, 10^6^:10^3^, 10^6^:10^2^, 10^6^:10^1^ and 10^6^:10^0^ CFU/mL, which were added to the 96-well plates and co-cultured for 48 hours at 37°C in an anaerobic environment. Thereafter, using the CV method, the biofilm forming capacity was ascertained.

### 2.3 Observation and measurement of the thickness of biofilm formation by confocal laser scanning microscopy

With some modifications, the Filmtracer Live/Dead biofilm viability kit (L10316, Thermo Fisher Scientific, United States) was used to stain the formed biofilms in accordance with the manufacturer’s instructions (He et al., 2021). After that, Confocal Laser Scanning Microscopy (CLSM) (NIKON ECLIPSE TI, Nikon, Tokyo, Japan) and imaging system (NIKON C2, Nikon, Tokyo, Japan) were used to measure the thickness of biofilm formation. In accordance to method 2.2.2, the samples were incubated anaerobically at 37°C for 48 hours with different bacterial volume ratios. After incubation, the samples were washed three times with sBHI. And then incubated with fluorescent stain, which was prepared by taking 3μL of propidium iodide stain and 3μL of SYTO^®^9 stain from the Filmtracer Live/Dead biofilm viability kit and adding them to 1mL of sBHI by avoiding light. The samples were then put in a 20×20 mm dish and 1 mL of sBHI was added to cover the surface by 2 mm (He et al., 2021). Claims were made using CLSM, and the excitation wavelengths for green and red light were 488 nm and 561 nm, respectively. In order to obtain a series of images of each layer and combine them, tomography was performed at intervals of 1 μm in the z-axis direction. For each sample, at least four fields of view were chosen in order to detect the thickness of the biofilm (Beaudoin et al., 2017; Ma et al., 2022).

### 2.4 Observation of biofilm formation by scanning electron microscopy

Similar to experimental method 2.3, different proportions of *G. vaginalis* and *E. coli* were co-cultured on specialized cell climbing glass (YA0350, Solarbio, Beijing, China) in 24-well plates at 37°C in an anaerobic environment for 48 hours. Biofilm formation was observed by scanning electron microscopy (SEM) (JSM-7900F, JEOL, Japan) at 5 kV after fixation with 1.5% glutaraldehyde (Bulacio et al., 2015; Niu et al., 2017).

### 2.5 Statistical analysis

Data was analyzed using SPSS version 26.0 (SPSS Statistics for Windows Version 26.0, IBM Corp, Armonk, NY, USA) by the unpaired t-test or non-parametric Wilcoxon signed-rank test. A *P* value < 0.05 was considered statistical significance.

## 3 Results

### 3.1 Collection of specimens

The *Gardnerella* strains included *G. vaginalis* ATCC14018 and five *Gardnerella* species strains (*G. vaginalis* 1-*G. vaginalis* 5) isolated from patients with BV. All *Gardnerella* strains were identified as *G. vaginalis* by 16S rRNA sequencing. *E. coli* ATCC25922 was identified as *E. coli*.

### 3.2 Biofilm formation

#### 3.2.1 Biofilm formation of *G. vaginalis* and *E. coli* by the CV method

As shown in **Table 1**, all strains of *G. vaginalis* exhibited the strongest biofilm forming capacity at 48 hours, which was stronger than the biofilm forming capacity at 24 hours and 72 hours, with a statistically significant difference (*P*<0.05). Additionally, *G vaginalis* ATCC14018 exhibited a stronger biofilm forming capacity at 48 hours compared to other strains, also with a statistically significant difference (*P*<0.05). Consequently, *G. vaginalis* ATCC14018 was chosen for further co-culture. The biofilm forming capacity of *E. coli* at 48 hours was stronger than that at 24 hours, and this difference was statistically significant (*P*<0.05), whereas the difference was not statistically significant (*P*>0.05) in contrast to the value after 72 hours. However, 48 hours was chosen as the co-culture observation period because the biofilm forming capacity was stronger at 48 hours than it was at 72 hours.

**Table 1:** Determination of biofilm forming capacity of *G. vaginalis* and *E. coli*.

Strains	Biofilm forming capacity at 24 hours (Mean ± SD)	Biofilm forming capacity at 48 hours (Mean ± SD)	Biofilm forming capacity at 72 hours (Mean ± SD)
*G. vaginalis* 1	1.15 ± 0.17^a^	3.79 ± 1.24^b^	2.04 ± 0.59^c^
*G. vaginalis* 2	1.01 ± 0.23^d^	4.04 ± 0.84^e^	2.27 ± 0.67^f^
*G. vaginalis* 3	0.92 ± 0.12^g^	3.43 ± 0.83^h^	1.97 ± 0.47^i^
*G. vaginalis* 4	1.10 ± 0.20^j^	4.07 ± 0.73^k^	1.96 ± 0.36^l^
*G. vaginalis* 5	1.04 ± 0.19^m^	3.63 ± 0.39^n^	1.79 ± 0.44°
*G. vaginalis* ATCC14018	1.17 ± 0.14^p^	5.72 ± 0.83^q^	2.40 ± 0.15^r^
*E. coli* ATCC25922	1.29 ± 0.13^s^	1.73 ± 0.17^t^	1.67 ± 0.45^u^

P_ab_ = 0.003; P_bc_ = 0.011; P_de_ = 0.000; P_ef_ = 0.002; P_gh_ = 0.000; P_hi_ = 0.004; P_jk_ = 0.000; P_kl_ = 0.000; P_mn_ = 0.000; P_no_ = 0.000; P_pq_ = 0.000; P_qr_ = 0.000; P_st_ = 0.001; P_tu_ = 0.743. (example: P_ab_ = 0.003 represents a statistically significant difference between a and b).

#### 3.2.2 Comparison of the biofilm forming capacity of *E. coli* in anaerobic and non-anaerobic environments

There was no statistically significant difference in the biofilm forming capacity of *E. coli* between an anaerobic environment and a 5% CO_2_ environment (*P* = 0.230, t = -1.277), which is commonly used for culturing *E. coli*. Therefore, the subsequent co-cultivation assay with *G. vaginalis* could be performed in an anaerobic environment.

#### 3.2.3 Comparison of the biofilm forming capacity of *G. vaginalis* alone, *E. coli* alone, and both co-cultured for 48 hours

**Figure 1:**
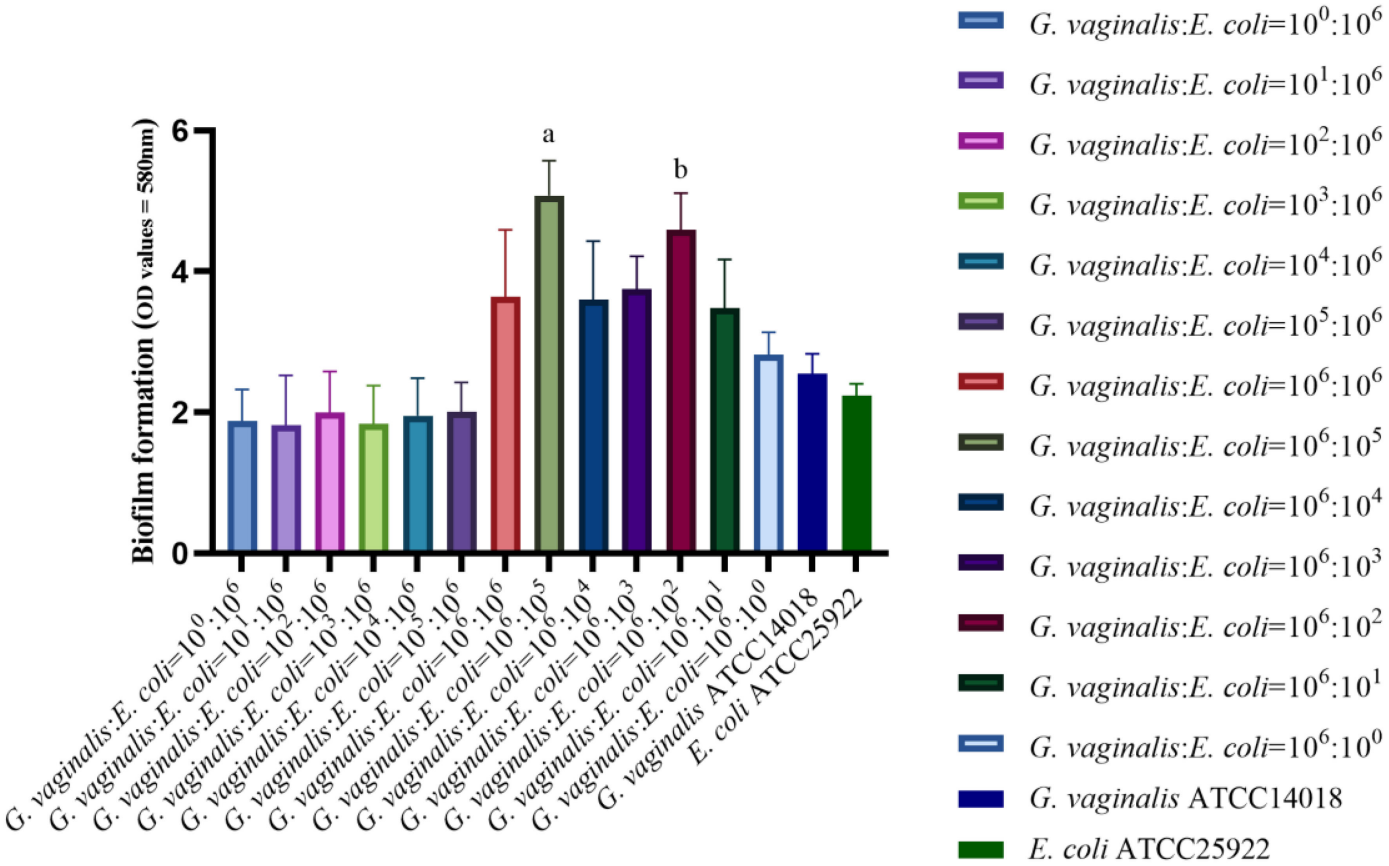
Biofilm forming capacity in co-culture of *G. vaginalis* and *E. coli* at different ratios. ^a^The biofilm forming capacity of *G. vaginalis* and *E. coli* at bacterial volume ratios of 10^6^:10^5^ CFU/mL is stronger than other ratios (*P*<0.05). ^b^The biofilm forming capacity of *G. vaginalis* and *E. coli* at bacterial volume ratios of 10^6^:10^2^ CFU/mL is stronger than other ratios except for 10^6^:10^5^ CFU/mL(*P*<0.05).

As demonstrated in **Figure 1**, the biofilm forming capacity of *G. vaginalis* and *E. coli* was strong at bacterial volume ratios of 10^6^:10^5^ CFU/mL and 10^6^:10^2^ CFU/mL, with statistically significant differences (*P*<0.01) when compared to all other ratios and with *G. vaginalis* and *E. coli* grown in isolation. The bacterial volume ratio of 10^6^:10^5^ CFU/mL resulted in a stronger biofilm forming capacity compared to 10^6^:10^2^ CFU/mL, and this difference was statistically significant (*P*<0.05). Therefore, a concentration of 10^6^:10^5^ CFU/mL was chosen for subsequent experiments.

### 3.3 Observation and measurement of the biofilm thickness by CLSM

According to the date in **Table 2**, the thickest biofilm was formed when the volume ratio of *G. vaginalis* to *E. coli* was 10^6^:10^5^ CFU/mL. This ratio resulted in a biofilm thickness as measured by CLSM that was significantly greater than all other ratios, except for 10^6^:10^2^ CFU/mL (*P*<0.05).

**Table 2:** Biofilm thickness in co-culture of *G. vaginalis* and *E. coli* at different ratios.

Strains	Biofilm thickness (μm)
*G. vaginalis*: *E. coli*=10^6^:10^0^	62.98 ± 15.568^a^
*G. vaginalis*: *E. coli*=10^6^:10^1^	65.63 ± 16.378^b^
*G. vaginalis*: *E. coli*=10^6^:10^2^	82.62 ± 14.565^c^
*G. vaginalis*: *E. coli*=10^6^:10^3^	54.64 ± 4.894^d^
*G. vaginalis*: *E. coli*=10^6^:10^4^	64.29 ± 17.310^e^
*G. vaginalis*: *E. coli*=10^6:^10^5^	94.22 ± 12.758^f^
*G. vaginalis*: *E. coli*=10^6^:10^6^	43.71 ± 5.223^g^
*G. vaginalis* ATCC14018	71.22 ± 6.827^h^
*E. coli* ATCC25922	60.75 ± 9.988^i^

P_af_ = 0.022; P_bf_ = 0.022; P_cf_ = 0.277; P_df_ = 0.000; P_ef_ = 0.032; P_gf_ = 0.000; P_hf_ = 0.010; P_if_ = 0.006; (example: P_af_ = 0.022 represents a statistically significant difference between a and f).

**Figure 2:**
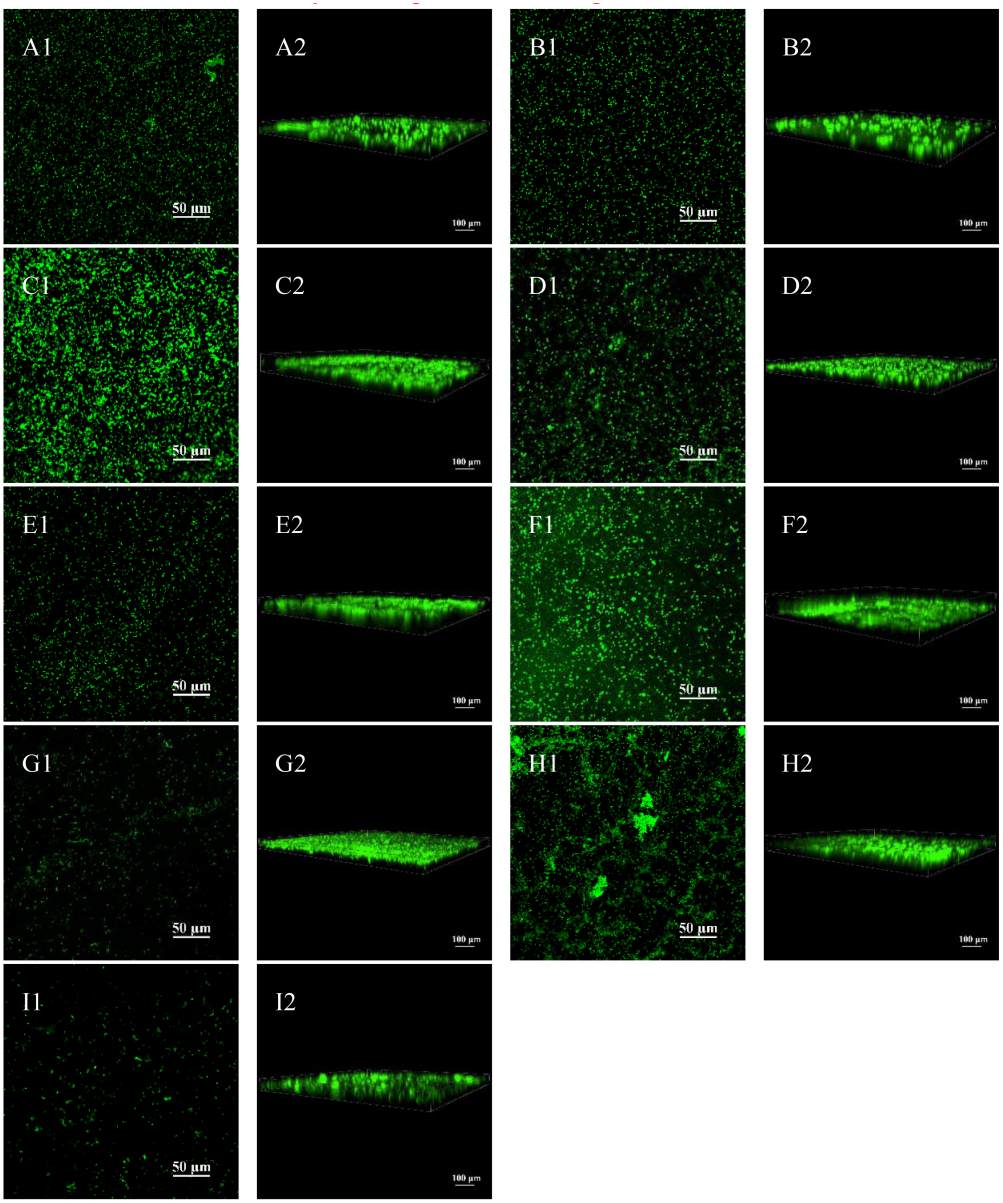
Biofilm formation of *G. vaginalis* and *E. coli* via CLSM. **(A1)** CLSM image at 10^6^:10^0^ CFU/mL; **(A2)** 3D image at 10^6^:10^0^ CFU/mL; **(B1)** CLSM image at 10^6^:10^1^ CFU/mL; **(B2)** 3D image at 10^6^:10^1^ CFU/mL; **(C1)** CLSM image at 10^6^:10^2^ CFU/mL; **(C2)** 3D image at 10^6^:10^2^ CFU/mL; **(D1)** CLSM image at 10^6^:10^3^ CFU/mL; **(D2)** 3D image at 10^6^:10^3^ CFU/mL; **(E1)** CLSM image at 10^6^:10^4^ CFU/mL; **(E2)** 3D image at 10^6^:10^4^ CFU/mL; **(F1)** CLSM image at 10^6^:10^5^ CFU/mL; **(F2)** 3D image at 10^6^:10^5^ CFU/mL; **(G1)** CLSM image at 10^6^:10^6^ CFU/mL; **(G2)** 3D image at 10^6^:10^0^ CFU/mL; **(H1)** CLSM image of cultured *G. vaginalis* only; **(H2)** 3D image of cultured *G. vaginalis* only; **(I1)** CLSM image of cultured *E. coli* only; **(I2)** 3D image of cultured *G. vaginalis* only.

**Figure 2** shows the performance and 3D images under CLSM after Live/Dead staining and it can be seen that there are more bacteria at the volume ratio of *G. vaginalis* to *E. coli* of 10^6^:10^5^ CFU/mL and 10^6^:10^2^ CFU/mL than the other ratio. We could also determine that the biofilm formed at 10^6^:10^5^ CFU/mL and 10^6^:10^2^ CFU/mL was the thickest by looking at the 3D image’s scale lines.

### 3.4 Observation of biofilm formation by SEM

**Figure 3:**
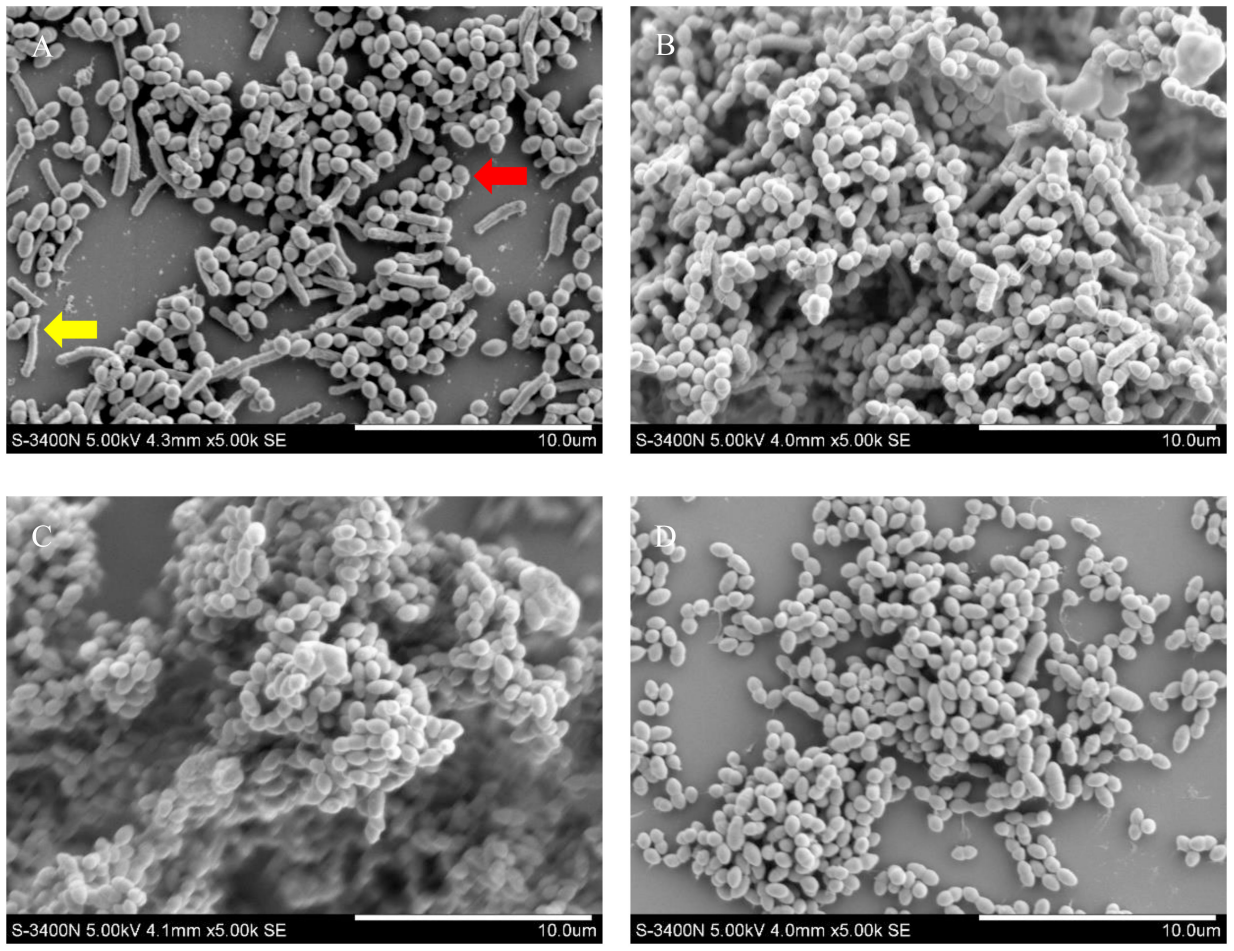
Biofilm formation of *G. vaginalis* and *E. coli* by SEM. **(A) **SEM image at a bacterial volume ratio of 10^6^:10^6^ CFU/mL; **(B) **SEM image at a bacterial volume ratio of 10^6^:10^5^ CFU/mL; **(C) **SEM image at a bacterial volume ratio of 10^6^:10^2^ CFU/mL; **(D) **SEM image cultured *G. vaginalis* only. Red arrow: *G. vaginalis*; yellow arrow: *E. coli*.

The number of bacteria and biofilm formation were higher when the volume ratio of *G. vaginalis* to *E. coli* was 10^6^:10^5^ CFU/mL and 10^6^:10^2^ CFU/mL (**Figures 3B, C**) than only *G. vaginalis* was cultured (**Figure 3D**). As shown in **Figure 3A**, despite both being rod-shaped, *E. coli* (yellow arrow) is longer than *G. vaginalis* (red arrow). Almost no *E. coli* was seen as demonstrated in **Figure 3C**.

## 4 Discussion

The incidence of mixed vaginitis has been increasing annually in recent years. It also makes diagnosing and treating the infection more difficult due to the combination of several pathogenic bacterial infections, unusual symptoms, and potential interactions between various vaginal pathogens. Imbalances in vaginal flora and biofilm formation have also been linked to an increased risk of infection with human immunodeficiency virus, herpes simplex type 2 and other sexually transmitted diseases like Chlamydia (MaChado et al., 2022). And it also increases the chances of recurrent infections. Our previous study involving 23,181 patients demonstrated that the most common mixed infection pattern was BV + AV in mixed vaginitis (Zong et al., 2023), which is consistent with previous findings (Fan et al., 2013). However, there are a few studies on mixed infection modes and the interaction mechanisms of multiple strains of mixed vaginitis (Benyas and Sobel, 2022; Xiao et al., 2022).

According to recent research, *Gardnerella* may cause BV through creating biofilms, promoting the attachment of other BV-associated pathogens, promoting the formation of biofilms in a wide range of microorganisms (Hardy et al., 2017; Jung et al., 2017), and generating pro-inflammatory cytokines and other virulence factors (Jang et al., 2017; Cerca, 2019; Shipitsyna et al., 2019). The interaction between a variety of microorganisms, including *Gardnerella*, is the basis of the pathogenesis of BV. The formation of biofilm is a key pathogenesis and drug resistance mechanism (Africa et al., 2014; Muzny et al., 2019; Castro et al., 2020). Anti-anaerobic medication penetration can be slowed down and bacterial tolerance to the environment can be increased by the physical barrier of a biofilm (Onderdonk et al., 2016). Meanwhile, the expression of the bacterial virulence factor cytolysin reduces the immune response and enhances chronic colonization in women (Castro et al., 2017). It can also reverse the vaginal microenvironment that is conducive to biofilm formation. Antimicrobial resistance has thus grown over the previous few decades and is still growing. There are some treatment options that are currently in use that are becoming less effective (Muñoz-Barreno et al., 2021). The interplay of various pathogen species, the role of biofilms, and whether or not it makes treatment more challenging are urgent problems that need to be solved for mixed vaginitis (Lohse et al., 2018).

AV is a vaginal inflammation that results in congestion and edema of the vaginal mucosa as well as the production of purulent secretions. It is caused by an increase in *E. coli*, *Streptococcus*, and other opportunistic pathogens with a decrease in or absence of *Lactobacillus*. The three main pathogenic bacteria are *Staphylococcus aureus*, *Streptococcus*, and *E. coli* (Tansarli et al., 2013; Rumyantseva et al., 2016; Donders et al., 2017). The development of biofilms contributes significantly to *E. coli*’s resistance to antibiotic invasion (Sharma et al., 2016). Furthermore, it has also been demonstrated that in urinary tract infection, the presence of *Gardnerella* induces the proliferation of *E. coli*, which plays an important role in urinary tract infection (Gilbert et al., 2017). Therefore, in this study, *G. vaginalis* as well as *E. coli* were co-cultured to observe biofilm formation and the interactions between various pathogens in order to create a model for further drug trials.

Biofilm formation is a continuous process of adhesion, coaggregation, maturation and dispersion (Jung et al., 2017). In this study, we measured the biofilm forming capacity of a reference strain of *G. vaginalis*, five *G. vaginalis* strains from BV patients, and an *E. coli* reference strain at various points in time. *G. vaginalis* and *E. coli* showed the greatest biofilm forming capacity at 48 hours. This indicated that the biofilm was maturing at 48 hours. These results are comparable to those of Castro et al (Castro et al., 2021). Qin et al. also discovered a similar outcome, namely that eight clinical strains of *Gardnerella* do not spontaneously dissolve during continuous cultivation and instead reach a steady biofilm formation *in vitro* at 48 hours (Qin et al., 2023). Using the CV method, we also observed that the biofilm forming capacity of *G. vaginalis* was greater after 48 hours than it after 24 hours. Meanwhile, the biofilm forming capacity of the reference strain of *G. vaginalis* was greater than that of the clinical strain as measured in this study. Because the most severe scenario of mixed vaginitis infection was simulated in this study using the combination with the strongest biofilm forming capacity in the co-culture, reference strains of *G. vaginalis* and *E. coli* were chosen and their co-cultivation was observed for 48 hours to see how their biofilms formed.

*G. vaginalis* is an anaerobic bacterium that is cultured under harsh conditions (Li et al., 2020), while *E. coli* is a facultative anaerobic bacterium, who is typically cultured in 5% CO_2_ environment (Blancarte-Lagunas et al., 2020). There are not any conclusive studies on whether its biofilm forming capacity is different in an anaerobic environment and a 5% CO_2_ environment. For the first time, we compared the biofilm forming capacity of *E. coli* in the two environments in this study, and found no statistically significant differences between the two environments. As a result, *G. vaginalis* and *E. coli* could be co-cultured in an anaerobic environment.

The most popular and broadly applicable technique for calculating the biofilm forming capacity is the CV method (Magana et al., 2018). However, some studies suggest that the CV method has a risk of unstable measurement, that the biofilm is easily removed by the rinsing process. Mixed infections with multiple pathogens make biofilm formation more complex (Røder et al., 2016; Magana et al., 2018). The ability to form biofilms cannot be determined solely by the CV method. Castro et al (Castro et al., 2022) co-cultured three BV-related anaerobic bacteria, *G. vaginalis*, *Atopobacterium*, and *Prevotella*, and compared biofilm formation using the CV method and fluorescence staining. Every method and culture time produced different results. Therefore, co-cultured biofilms should not be subjected to the CV method alone as an accurate quantitative method. In addition to the CV method, biofilm formation in *G vaginalis* and *E coli* co-culture biofilm model was evaluated in this study in a number of ways using biofilm thickness measurement under CLSM following Live/Dead staining, pathogenic bacteria observation, and biofilm morphology analysis under SEM. The benefits of CLSM after Live/Dead staining include clear imaging, the capacity to asses bacterial viability (Freitas et al., 2014; Beaudoin et al., 2017), and the capacity to measure biofilm thickness in the Z-axis direction (He et al., 2021), all of which help to further minimize errors brought on by the use of the CV method. In our study, the volume ratio of 10^6^:10^5^ CFU/mL for *G. vaginalis* and *E. coli* produced the thickest biofilm and differed statistically from the other concentrations, with the exception of 10^6^:10^2^ CFU/mL. Biofilm formation was greater under SEM when the ratio of *G. vaginalis* to *E. coli* was 10^6^:10^5^ CFU/mL and 10^6^:10^2^ CFU/mL. This was the same as the biofilm forming capacity measured by the CV method and biofilm thickness measured by CLSM. This study verified that, at a ratio of 10^6^:10^5^ CFU/mL, the biofilm forming capacity of *G. vaginalis* and *E. coli* was superior to that of other ratios, *G. vaginalis* by itself, and *E. coli* alone, employing a variety of techniques like the CV method, measurement of biofilm thickness by CLSM after Live/Dead staining, and SEM observation. Thus, future research on mixed BV and AV infections should take this ratio as a model.

However, as the amount of *E. coli* decreased, no obvious *E. coli* was observed under SEM. Actually, *E coli* was essentially absent when the ratio of *G vaginalis* to *E coli* was 10^6^:10^3^ CFU/mL by SEM. On the other hand, the biofilm forming capacity peaked at a bacterial biomass ratio of 10^6^:10^2^ CFU/mL and 10^6^:10^5^ CFU/mL. Research has shown that 92% of *Atopobium* lost viability when incubated alone for 48 hours without *G vaginalis*, but that viability increased when *Atopobium* was co-cultured with *G vaginalis* or after being treated with sterile supernatants of *G vaginalis* (Castro et al., 2020). According to an *in vitro* study, *Staphylococcus aureus* could infiltrate deep host tissues and contribute to the pathogenic process through the hyphae that carry adherent *Candida* (Schlecht et al., 2015). Meanwhile, research has demonstrated that the biofilm that *Candida* forms could shield mixed anaerobic bacteria, enabling them to grow in an oxygen environment (Fox et al., 2014). And mutual promotion of proliferation between *E. coli* and *Streptococcus* had been demonstrated in a cross-sectional study (Cools et al., 2016). Furthermore, a strong inhibitory effect on the biofilm of *G. vaginalis* has been shown for the supernatant of a co-culture of *Enterococcus faecalis* and *Lactobacillus* (Zhang et al., 2023). More research is needed to determine whether *E. coli* acts similarly, whether its products can encourage the formation of *Gardnerella* biofilms, and the precise mechanism of action.

In conclusion, the ideal ratio of biofilm formation in mixed infections of *G. vaginalis* and *E. coli* was identified. This information can be applied to future research on mixed infections of BV and AV and their drug susceptibility. However, this study was limited to *in vitro* experiments with only two species, *G. vaginalis* and *E. coli*. Moreover, as BV and AV are linked to numerous other related bacteria, more research is required to create a mixed infection model of BV and AV in the future. Additionally, more research in the intricate vaginal microenvironment is required to figure out the best way to give medications and increase mixed vaginitis’s effectiveness.

## Data availability statement

The original contributions presented in the study are included in the article/supplementary material. Further inquiries can be directed to the corresponding author.

## Ethics statement

Approval was granted by the Ethics Committee of Beijing Obstetrics and Gynecology Hospital, Capital Medical University (No.2022-KY-064-01). The studies were conducted in accordance with the local legislation and institutional requirements. The participants provided their written informed consent to participate in this study.

## Author contributions

XS: Conceptualization, Data curation, Investigation, Methodology, Validation, Writing – original draft, Writing – review & editing. HB: Data curation, Methodology, Validation, Writing – original draft. LF: Project administration, Supervision, Validation, Writing – review & editing. XZ: Data curation, Investigation, Writing – original draft. XWZ: Data curation, Investigation, Writing – original draft. ZL: Funding acquisition, Methodology, Project administration, Resources, Supervision, Writing – review & editing.
